# Prognostic Stratification of Epithelioid Pleural Mesothelioma Based on the Hippo-TEADs Network

**DOI:** 10.3390/cancers17030469

**Published:** 2025-01-30

**Authors:** Anello Marcello Poma, Rossella Bruno, Iacopo Petrini, Iosè Di Stefano, Alessandra Celi, Andrea Sbrana, Sabrina Cappelli, Antonio Chella, Franca Melfi, Marco Lucchi, Greta Alì

**Affiliations:** 1 Department of Surgical, Medical, Molecular Pathology and Critical Area, University of Pisa, 56126 Pisa, Italy; iose@outlook.it (I.D.S.); aled.celi1@gmail.com (A.C.); franca.melfi@unipi.it (F.M.); marco.lucchi@unipi.it (M.L.); greta.ali@unipi.it (G.A.); 2 Unit of Pathological Anatomy, University Hospital of Pisa, 56126 Pisa, Italy; rossella.bruno@for.unipi.it; 3 Department of Translational Research and of New Surgical and Medical Technologies, University of Pisa, 56126 Pisa, Italy; iacopo.petrini@unipi.it; 4 Unit of Pneumology, University Hospital of Pisa, 56124 Pisa, Italy; andrea.sbrana@ao-pisa.toscana.it (A.S.); sabrina.cap@tiscali.it (S.C.); anto.kell@tiscali.it (A.C.)

**Keywords:** pleural mesothelioma, Hippo pathway, YAP1, TAZ, TEADs, prognosis

## Abstract

*Background.* The Hippo pathway is the most frequently altered signaling in pleural mesothelioma (PM). Epithelioid PM (ePM) is associated with better outcome than non-epithelioid subtypes, but its prognosis can be heterogeneous. Here, we tried to stratify ePM using the expression levels of the Hippo-TEAD network. *Methods.* Thirty patients with ePM were included in this study. Tumors were stratified using the expression levels of 74 genes belonging to the Hippo-TEAD network and using the non-negative matrix factorization algorithm. Results were validated using ePM cases from the TCGA cohort. Alterations associated with the molecular subgroups were investigated using mutation and copy number alteration data from TCGA. *Results.* Two groups of ePM (i.e., HP1 and HP2) were identified and validated using TCGA data. HP2 comprises about one-third of tumors. These tumors are frequently high-grade (73% vs. 35%), have higher levels of downstream Hippo effectors (i.e., *YAP1*, *WWTR1* and *TEADs*), lower levels of *VSIR*—which encodes for VISTA—and poorer PFS and OS. HP2 tumors commonly harbor homodeletions in Hippo core suppressors (25% vs. 3%), while no specific gene mutation or copy number alterations of Hippo genes was associated with the two groups. *Conclusions.* ePM can be stratified in prognostic subtypes based on the expression levels of the Hippo-TEAD network. Higher levels of downstream Hippo effectors are associated with poor response to platinum-pemetrexed doublet and worse OS. The stratification of ePM based on the activation of the YAP/TAZ-TEAD axis is an intriguing approach in the light of the inhibitors of this signaling that are currently under investigation.

## Simple Summary

The Hippo pathway is the most commonly altered signaling in pleural mesothelioma (PM). The aim of our retrospective study was to stratify patients with epithelioid PM using the expression levels of the Hippo-TEAD network. We identified two groups of tumors; HP2 tumors, which are about one-third of the total, have higher levels of downstream Hippo oncogenes, lower mRNA levels of the immune checkpoint VISTA, and poorer progression-free and overall survival. These tumors often harbor homodeletion of Hippo core suppressors.

## 1. Introduction

Pleural mesothelioma (PM) is a lethal cancer originating from pleural cells and associated with asbestos exposure [1]. GLOBOCAN 2022 estimates reported 30,618 new cases of PM and 25,372 PM-related deaths worldwide, accounting for 0.2 and 0.3% of all new cancer diagnoses and cancer-related deaths [2]. The World Health Organization (WHO) classification of tumors of the pleura defines three histological subtypes—epithelioid (ePM), biphasic, and sarcomatoid—which are characterized by different but poor prognosis [3]. Despite being the subtype that generally has the better outcome, ePM is clinically heterogeneous. Hence, an adequate stratification of patients with ePM is crucial for clinical management. To that aim, the latest WHO classification has introduced nuclear grading for ePM. This approach is based on the evaluation of nuclear atypia, mitotic count, and necrosis to identify high-grade tumors that have a poorer overall survival [3].

The treatment of PM has undergone very little improvements for several years. It is still based—when feasible—on a multimodality approach with surgery and platinum-pemetrexed chemotherapy, but the overall efficacy is limited [4]. After the results of the CheckMate 743 trial, first-line nivolumab plus ipilimumab was implemented in the management of PM. However, patients with epithelioid histology showed no significant overall survival improvement compared to the chemotherapy group [5].

From a molecular point of view, The Cancer Genome Atlas consortium has paved the way for the discovery of prognostic biomarkers and potential therapeutic targets in PM, which still lacks effective treatment options [6]. Large-scale sequencing studies have identified the Hippo pathway as the top recurrently altered signaling in PM [6,7]. This pathway is composed by a core kinase module, which responds to several stimuli, especially mechanical cues. The kinase module, which acts as an oncosuppressor, is composed primarily of STK3/4 and LATS1/2 with the respective scaffold proteins. The output of the kinase module is the phosphorylation and consequent inactivation of the oncoproteins YAP and TAZ. Unphosphorylated YAP and TAZ bind together and trigger the transcription mediated by TEA domain transcription factors (TEADs) [8].

The activation of the YAP/TAZ-TEAD axis is associated with poor prognosis in several cancer types [9,10]. Hence, blocking this axis is an appealing strategy, especially in PM and other tumors harboring Hippo pathway genetic alterations or YAP/TAZ hyperactivation. However, the use of systemic drugs inhibiting the YAP/TAZ-TEAD axis poses great challenges due to potential side effects. In fact, the activation of YAP/TAZ-TEAD is essential in some normal cells, especially those with high regenerative potential, and it may even have tumor suppressor activity in selected cancers. The first generation of YAP/TAZ inhibitors had multi-target activity, thus further complicating the control of side effects. The new generation of drugs blocking this axis often targets TEAD palmytoilation or the interaction between TEADs and the YAP/TAZ complex. While these new drugs showed promising preclinical results in terms of target specificity and toxicity profiles, tumor elimination was not complete, and tumor regrowth was observed [11,12]. The first clinical data regarding the inhibition of YAP/TAZ-TEAD axis were reported for the drug VT-3989 in 2023 (NCT04665206). In this trial, which included 69 patients with refractory tumors (43 with mesothelioma), 7 patients achieved a partial response while the others showed stable disease [13]. Other trials are currently under investigation and will provide useful data for this new generation of drugs [13,14,15,16].

Here we tried to stratify ePM in prognostic subtypes based on the expression levels of the extended Hippo-TEAD network.

## 2. Materials and Methods

### 2.1. Patients

A total of 30 patients affected by ePM were included in the study. All cases were diagnosed at the University Hospital of Pisa between 2012 and 2019. The samples were collected before starting any treatment. All tumors underwent histological revision according to the 2021 WHO guidelines [3]. All patients were treated with cisplatin or carboplatin plus pemetrexed. Progression-free (PFS) and overall survival (OS) were defined as time in months between the start of treatment and the first evidence of tumor progression and death, respectively. In the absence of adverse events, patients were right-censored at the last follow-up visit. Tumor progression was defined as an increase in size or the occurrence of new tumor foci. The study was approved by the local Ethics Committee (protocol code ANEMONE, approval date 1 August 2023).

### 2.2. Gene Expression Assay

White sections of 5 µm thick formalin-fixed paraffin-embedded (FFPE) obtained from biopsy or surgical specimens were used for RNA isolation using the RNeasy FFPE kit (Qiagen, Hilden, Germany). RNA concentration and quality was assessed by spectrophotometry (Trinean, Gentbrugge, Belgium). The gene expression assay was performed on the nCounter platform (nanoString Technologies, Seattle, WA, USA) and using a custom panel including 88 genes (Supplementary Table S1); 74 belonged to the Hippo-TEAD network, 4 were immune-related genes, and 10 were housekeeping genes used for normalization. For the assay, 150 ng of RNA was hybridized with capture and reported probes at 65 °C for 20 h. Digital counting of target signals was performed at maximum sensitivity (i.e., 555 fields of view).

### 2.3. Immunohistochemistry (IHC) and Fluorescence In Situ Hybridization (FISH)

For BAP1 IHC evaluation, 4 µm thick FFPE sections were deparaffinized and rehydrated in ethanol solutions. Staining was carried out on an automated stainer (Ventana Medical System, Oro Valley, AZ, USA) using a mouse monoclonal antibody (clone C-4, 1:100 dilution, Santa Cruz Biotechnology, Dallas, TX, USA) and the UltraView DAB IHC detection kit (Ventana Medical System). Counterstaining with hematoxylin was performed. BAP1 was considered to be expressed in the presence of unambiguous mesothelial cell nuclei staining without intensity or extension cut-offs. In negative controls, the primary antibody was omitted. The internal positive control for each sample was represented by non-mesothelial cells expressing nuclear BAP1.

FISH was used to evaluate *CDKN2A* (*p16*) deletion. The Vysis LSI *p16* (spectrum orange) and CEP9 (spectrum green) kit (Abbott Molecular, Des Plaines, IL, USA) were used as previously described [17]. Homozygous deletion of *p16* was defined as loss of both orange signals in more than 11% of tumor nuclei showing CEP9 green signals. At least 60 non-overlapping and well-defined cells were evaluated per sample.

### 2.4. Data Analysis and Statistics

Raw counts were normalized according to standard nCounter procedures and using the nSolver Analysis Software v.4.0. Briefly, background subtraction was first applied to raw counts; then, a two-step normalization was performed to adjust potential differences due to hybridization efficiency and RNA input. Normalized counts were log2-transformed for downstream analyses. The 74 genes belonging to the Hippo pathway were used to group samples by the non-negative matrix factorization algorithm and following the procedures of NMF R package v.0.27. Brunet’s method and 100 runs were set to identify the best factorization rank; ranks from 2 to 6 were tested. The optimal rank was chosen according to the highest cophenetic coefficient. Suppression and onco scores were computed by averaging the expression levels of the core genes [9]. In detail, the genes considered for the onco score were *TEAD1*, *TEAD2*, *TEAD3*, *TEAD4*, *YAP1*, and *WWTR1*, while the genes used to compute the suppression score were *FRMD6*, *LATS1*, *LATS2*, *MOB1A*, *MOB1B*, *NF2*, *SAV1*, *STK3*, *STK4*, *TAOK1*, *TAOK2*, *TAOK3*, and *WWC1*. The ratio score was obtained by dividing the onco score by the suppression score. Survival curves were built using the Kaplan–Meier method, and differences were tested by the log-rank test. TCGA data of PM were retrieved from cBioPortal [18]. In detail, clinical, transcriptome, mutation, copy number alteration (CNA), and reverse-phase protein microarray data were matched by tumor sample barcode and used. Data from non-epithelioid tumors were not further considered. Level 3 transcriptome data from TCGA were log2-transformed after adding 1 to all gene counts to avoid dealing with log2 of 0. Mutation data from TCGA were analyzed using maftools Bioconductor package v.2.20.0. The correlation between continuous variables was tested using Pearson’s method. Continuous variables are presented as median and interquartile range (IQR), and analyzed by the Wilcox test. The association between categorical variables was assessed using either the chi-square or Fisher’s exact test. *p*-values of 0.05 were deemed significant. All analyses were performed in the R environment (https://www.r-project.org/, v.4.3.3, last accessed on 25 June 2024).

## 3. Results

### 3.1. Identification of Two Prognosis-Related Groups

**Figure 1:**
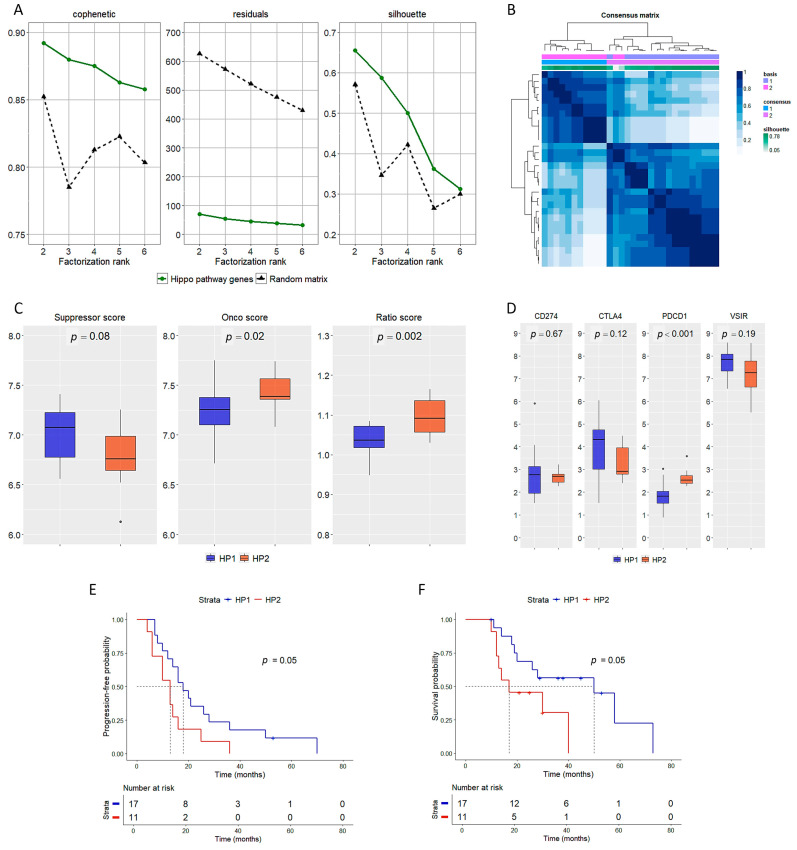
Identification of two subgroups of eMPM based on the expression levels of the Hippo-TEADs network. (**A**) A factorization rank of 2 (i.e., two groups) was the best fit obtained by the non-negative matrix factorization algorithm. Here the cophenetic coefficient (i.e., a measure of the stability of the clusters), the residuals (i.e., variability not explained by the model), and the silhouette (i.e., average goodness of fit of each sample within its cluster) are shown. (**B**) Consensus matrix obtained with the best factorization rank (*n* = 2). Samples are clustered by similarity, and the shades-of-green sidebar shows the silhouette of each sample in relation to the consensus cluster. (**C**) Comparison of suppressor, onco, and ratio score between HP1 and HP2. (**D**) Expression of immune-related genes in HP1 and HP2. Progression-free (**E**) and overall survival (**F**) of HP1 and HP2 patients.

The optimal factorization rank according to the NMF algorithm was 2 (Figure 1A); the two groups are henceforward defined as HP1 (*n* = 17) and HP2 (*n* = 11). Two samples had a poor silhouette (i.e., <0.1) as shown in the consensus map (Figure 1B) and were excluded from further analyses. The clinical-pathological features of HP1 and HP2 are reported in Table 1. HP2 had a higher onco and ratio score, and a lower suppression score, though the latter was not significant (Figure 1C). Among immune-related genes, *VSIR*—which encodes for VISTA protein—was the only one abundantly expressed; *VSIR* levels were higher in HP1, though not significantly. *PDCD1* expression was higher in HP2 than in HP1, but the mRNA levels were still poor (Figure 1D). Patients of the HP1 group had a longer PFS (18 [95% CI 10–28] vs. 13 [95% CI 11–16] months, Figure 1E) and OS (50 [95% CI 10-not reached] vs. 17 [95% CI 8-not reached] months, Figure 1F) than HP2 patients.

### 3.2. Validation of the Two Prognosis-Related Groups Using TCGA Data

**Figure 2:**
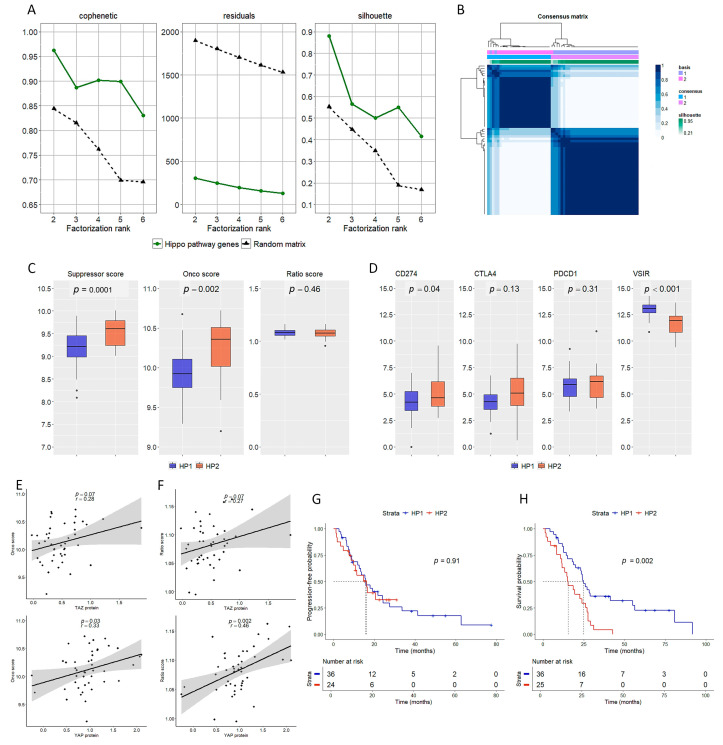
Validation of the two subgroups of ePM using TCGA data. (**A**) Using the same set of genes, ePM from TCGA showed an optimal clustering with a factorization rank of 2. (**B**) Consensus matrix obtained with rank = 2. (**C**) Suppression, onco, and ratio scores of HP1 and HP2 in the TCGA cohort. (**D**) Expression of 4 immune-related genes in ePM from the TCGA cohort. (**E**) Correlation between onco score and YAP and TAZ proteins. (**F**) Correlation between ratio score and YAP and TAZ proteins. Progression-free (**G**) and overall survival (**H**) of HP1 and HP2 patients from the TCGA cohort.

The 74-gene set belonging to the Hippo pathway and used in the previous phase was retrieved from transcriptomic data of the PM cohort of TCGA. Non-epithelioid cases were excluded. Again, the NMF algorithm identified two groups as optimal rank (Figure 2A). The consensus map shows the separation in two groups (i.e., HP1 [*n* = 36] and HP2 [*n* = 24], Figure 2B). HP2 had higher onco and suppressor scores, while no differences were observed in terms of ratio score (Figure 2C). Consistent with the previous findings, *VSIR* was the most expressed immune-related gene; *VSIR* levels were significantly more abundant in HP1. On the other hand, *CD274*—encoding for PD-L1—was more expressed in HP2 (Figure 2D). Then, we correlated the mRNA scores with protein levels of YAP and TAZ. Both onco and ratio scores positively correlated with YAP protein levels. Similarly, both scores positively correlated with TAZ protein, though without reaching statistical significance (Figure 2E,F). Finally, patients belonging to the HP2 group had a worse overall survival (15.4 [95% CI 10.8–23.9] vs. 24.9 [95% CI 18.8–41.5] months, Figure 2H) than those of HP1. No significant differences in terms of PFS were observed (Figure 2G).

### 3.3. Exploring Molecular Alterations Associated with HP1 and HP2

**Figure 3:**
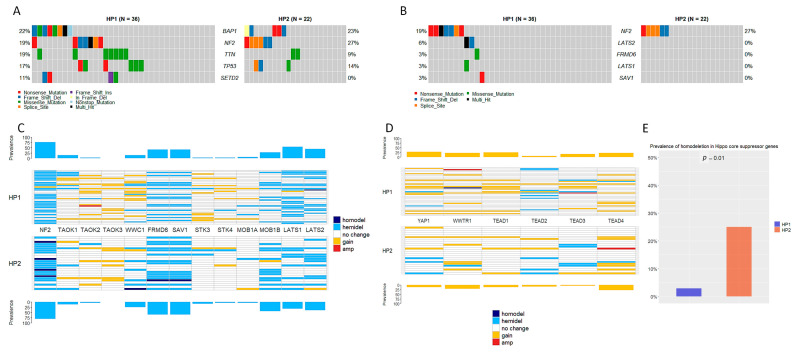
Gene mutations and copy number alterations in HP1 and HP2. (**A**) The oncoplot shows the top mutated genes in ePM with no differences between HP1 and HP2. (**B**) Hippo core suppressors other than *NF2* are rarely mutated in ePM. (**C**) Copy number alterations of Hippo core suppressors in HP1 and HP2. (**D**) Copy number alterations of downstream Hippo effectors in HP1 and HP2. (**E**) Different prevalence of homodeletions in Hippo core suppressor genes in HP1 and HP2.

HP1 and HP2 were not associated with specific gene mutations. Among the top mutated genes, a similar prevalence of mutation was observed for *BAP1*, *NF2*, and *TP53*. *SETD2* mutations (*n* = 4) were observed in HP1 only (Figure 3A). No mutations were observed in the genes of the onco score (i.e., *YAP1*, *WWTR1*, *TEAD1*, *TEAD2*, *TEAD3*, *TEAD4*), while mutations in suppressor genes—other than *NF2*—of the core Hippo pathway were observed at very low prevalence in HP1 only (Figure 3B). Hemideletions in Hippo core suppressors were common but similarly prevalent in HP1 and HP2 (Figure 3C), as well as copy number gain of oncogenes (Figure 3D). Notably, tumors belonging to the HP2 group harbored homodeletions in at least one Hippo core suppressor gene more frequently than those in HP1 (6 [25%] vs. 1 [3%], Figure 3E).

## 4. Discussion

PM has been defined as an orphan disease due to the lack of effective treatments and also to the inaccurate stratification of patients [19]. Indeed, histological subtyping provides the most effective prognostic stratification [20,21], but molecular data can predict the clinical outcome [22,23,24]. Molecular stratification might be useful, especially in ePM, which can display very heterogeneous outcomes [25,26].

Since the TCGA breakthrough [6], it has become evident that the Hippo pathway is the better candidate to providing solutions for improving MPM management. Indeed, the regulation of the Hippo pathway is complex and not always straightforward [27], but the selective inhibition of downstream Hippo effectors is an appealing strategy in PM [8]. Currently, there are four ongoing phase I or II clinical trials investigating the safety and biological activity of different inhibitors. Three of them work by inhibiting TEAD palmitoylation (NCT04665206 [13], NCT06566079 [15], and NCT06251310 [16]), and one disrupts the interaction of YAP/TAZ and TEADs (NCT04857372, [14])

Here we used the expression levels of the extended Hippo-TEAD network to stratify ePM. We identified two groups (i.e., HP1 and HP2), with HP2 characterized by high mRNA levels of downstream effectors of the Hippo pathway (i.e., *YAP1*, *WWTR1*, and *TEADs*) and poor OS. In the presence of a homogeneous type of systemic therapy, PFS was also poorer in HP2, thus indicating that these tumors might be less responsive to the standard platinum-pemetrexed doublet. Interestingly, the HP2 group had a double rate of high-grade tumors (73% vs. 35%), coherent with the prognostic impact of nuclear grading.

Of note, the expression levels of the suppression score were not consistent between the original and the validation cohorts. Since Hippo core suppressors harbor alterations more frequently than downstream Hippo effectors, a different prevalence of aberrations or even feedback mechanisms might be accountable for this controversial findings [9]. Indeed, *YAP1*, *WWTR1*, and *TEAD* genes are rarely altered—especially in non-squamous malignancies [9]—and might provide more stable results. The discrepancy of the suppressor score between original and validation cohort might also be due to the different techniques used (i.e., nCounter versus RNA-seq). On the one hand, a hybridization-based approach might have lost some mRNA isoforms; on the other hand, a sequencing approach might inflate the mRNA levels with inactive isoforms.

It might be of interest to note that TEAD isoforms in ePM cases from TCGA harbored both copy gains and deletions, with *TEAD4* being the most prone to copy gain.

We also confirmed that *VSIR*, which encodes for VISTA, is the immune checkpoint mostly expressed in ePM, though in our cohort only a limited number of genes were evaluated. *VSIR* levels were higher in HP1, though not significantly in our original cohort, probably due to the limited sample size. Nevertheless, higher levels of *VSIR* in HP1 are consistent with the association of this immune checkpoint with a better OS observed in solid malignancies including PM [28,29]. The mRNA levels of *CTLA4*, *CD274* (encoding for PD-L1), and *PDCD1* (encoding for PD1) were very low, thus making any difference observed of poor relevance. Given the low concordance of immune checkpoint levels—apart from *VSIR*—in HP1 and HP2 observed in the original and the TCGA cohort, immune aspects were not further explored.

Next, we sought to identify molecular alterations supporting the distinction of the two groups. None of the top mutated genes nor Hippo core gene mutations were associated with HP1 and HP2. Similarly, the hemideletion of Hippo suppressors and copy number gain of onco score genes were evenly distributed. On the other hand, homodeletion of Hippo core suppressors was more prevalent in HP2 (25% vs. 3%). Indeed, these findings do not explain the different mRNA level of the core genes used in the scores, but they are coherent with the silencing of the Hippo pathway and the consequent activation of the YAP/TAZ-TEAD axis. Epigenetic mechanisms might be accountable for the different mRNA levels between HP1 and HP2, as hypothesized for tumors showing a strong association of the pathway with survival but a low rate of DNA aberrations in these genes [9].

There are some limitations that should be acknowledged. First, the sample size of the original cohort is small but coherent with the rarity of the tumor. On the other hand, we reported a mono-institutional cohort of patients with ePM, which were treated similarly. Also, the findings were validated using a publicly available dataset. Second, immune data were not completely consistent between the original cohort and the TCGA validation set. The only exception is the expression of *VSIR*, which is the most expressed immune-related gene in ePM and is associated with better prognosis. Nevertheless, the relationships between the Hippo pathway and the immune profile in ePM cannot be derived from the present study and deserve further investigation. Of note, the surgical rate of our cohort (i.e., about 64%) is higher than in PM patients, though in line with some reports on selected cases [30]. Indeed, the small sample size and the surveillance program for individuals exposed to asbestos at our institution might be accountable for the high rate of surgery. While there is no complete agreement about the impact of different types of surgery and number of chemotherapy cycles on overall survival, these aspects were not evaluated in our study, and their influence on the results cannot be excluded.

## 5. Conclusions

We demonstrated the existence of two molecular groups of ePM based on the expression levels of the extended Hippo-TEAD network. The groups (i.e., HP1 and HP2) exist in a proportion of about 1.5:1, which was consistent in the original and in the validation set. HP2 tumors are characterized by high levels of downstream effectors of the Hippo pathway, lower levels of *VSIR*, and poorer outcomes. The stratification of patients based on the activation of the YAP/TAZ-TEAD axis might also be meaningful in light of the new inhibitors of this signaling that are being tested in clinical and pre-clinical studies. Further studies should investigate genetic and transcriptomic signatures that might predict the therapeutic response of YAP/TAZ-TEAD inhibitors.

## Figures and Tables

**Table 1:** Clinical-pathological characteristics of HP1 and HP2 ePM.

Variable		HP1 (*n* = 17)	HP2 (*n* = 11)	*p*-Value
Sex, male	*n* (%)	10 (59%)	7 (64%)	1
Age, years	median (IQR)	65 (63–75)	70 (65–75)	0.32
Surgery, yes	*n* (%)	12 (71%)	6 (55%)	0.44
Asbestos exposure, yes ^a^	*n* (%)	8 (50%)	7 (64%)	0.76
Smoking status, never ^b^	*n* (%)	4 (40%)	4 (67%)	0.61
ECOG PS, 2	*n* (%)	2 (12%)	5 (45%)	0.07
Grade, high-grade	*n* (%)	6 (35%)	8 (73%)	0.12
Tumor extension, T3 or T4 ^c^	*n* (%)	10 (71%)	7 (64%)	1
Lymph node involvement, yes ^d^	*n* (%)	6 (50%)	7 (67%)	0.66
Stage, III-IV ^e^	*n* (%)	8 (57%)	8 (73%)	0.68
BAP1 IHC expression, lost ^f^	*n* (%)	8 (80%)	4 (67%)	0.60
p16 FISH status, deleted ^g^	*n* (%)	4 (57%)	2 (33%)	0.59

ECOG PS, Eastern Cooperative Oncology Group Performance Status; IHC, immunohistochemistry; FISH, fluorescence in situ hybridization. ^a^ not available for 1 patient of HP1. ^b^ not available for 7 patients of HP1 and 5 patients of HP2. ^c^ not available for 3 patients in HP1. ^d^ not available for 5 patients in HP1 and 2 patients in HP2. All positive cases but one are N2. ^e^ not available for 3 patients in HP1. ^f^ not available for 7 HP1 and 5 HP2 patients. ^g^ not available for 10 HP1 and 5 HP2 patients.

## Data Availability

The datasets generated and/or analyzed during the current study are available from the corresponding author on reasonable request.

## Supplementary Materials

The following supporting information can be downloaded at: https://www.mdpi.com/article/10.3390/cancers17030469/s1, Table S1: List of genes analyzed by the nCounter custom panel.
